# Roles, outcomes, and enablers within research partnerships: A rapid review of the literature on patient and public involvement and engagement in health research

**DOI:** 10.1186/s40900-023-00448-z

**Published:** 2023-06-15

**Authors:** Anne Wettergren Karlsson, Anne Kragh-Sørensen, Kirsten Børgesen, Karsten Erik Behrens, Torben Andersen, Maiken Langhoff Kidholm, Mette Juel Rothmann, Marjolijn Ketelaar, Astrid Janssens

**Affiliations:** 1 Department of Public Health, User Perspectives and Community-Based Interventions, University of Southern Denmark, Odense, Denmark; 2 Steno Diabetes Center Odense, Odense University Hospital, Odense, Denmark; 3 Center for Innovative Medical Innovation, Odense University Hospital, Odense, Denmark; 4 Department of Clinical Research, University of Southern Denmark, Odense, Denmark; 5 Center of Excellence for Rehabilitation Medicine, Brain Center, University Medical Center Utrecht, Utrecht, The Netherlands; 6 Center of Excellence for Rehabilitation Medicine, De Hoogstraat Rehabilitation, Utrecht, The Netherlands; 7 Bioethics and Health Humanities, Julius Center for Health Sciences and Primary Care, University Medical Center Utrecht, University Utrecht, Heidelberglaan 100, 3584 CX Utrecht, The Netherlands; 8 Centre for Research with Patients and Relatives, Odense University Hospital, Odense, Denmark; 9 University of Exeter Medical School, Exeter, UK

**Keywords:** Patient and public involvement, Coproduction, Partnerships, Evaluation, Rapid review, Co-authorship, Health research

## Abstract

**Background:**

Recent studies mention a need to investigate partnership roles and dynamics within patient and public involvement and engagement (PPIE) in health research, and how impact and outcomes are achieved. Many labels exist to describe involvement processes, but it is unknown whether the label has implications on partnerships and outcomes. This rapid review investigates how roles between patients, relatives and researchers in a broad variety of PPIE activities in health research are described in peer reviewed papers and explores what enables these partnerships.

**Methods:**

Rapid review of articles published between 2012 and February 2022 describing, evaluating, or reflecting on experiences of PPIE in health research. All research disciplines and research areas were eligible. Four databases (Medline, Embase, PsychInfo and CINAHL) were searched between November 2021 and February 2022. We followed PRISMA guidelines and extracted descriptive factors: year, origin, research area and discipline, study focus, framework used and co-authorship. On a selection of articles, we performed a narrative analysis of partnership roles using Smits et al.’s. Involvement Matrix. Lastly, we performed a meta synthesis of reported enablers and outcomes of the partnerships. Patients and Relatives (PRs) have been involved in the whole rapid review process and are co-authors of this article.

**Results:**

Seventy articles from various research disciplines and areas were included. Forty articles were selected for a narrative analysis of the role description of PRs and researchers, and a meta synthesis of enablers and outcomes. Most articles described researchers as decision-makers throughout the research cycle. PRs most often were partners when they were included as co-authors; they were mostly partners in the design, analysis, write-up, and dissemination stages. Enablers of partnerships included: PR training, personality of PRs and communication skills, trust, remuneration and time.

**Conclusions:**

Researchers’ decision-making roles gives them control of where and when to include PRs in their projects. Co-authorship is a way of acknowledging patients’ contributions which may lead to legitimation of their knowledge and the partnership. Authors describe common enablers, which can help future partnership formation.

**Supplementary Information:**

The online version contains supplementary material available at 10.1186/s40900-023-00448-z.

## Abstract (plain language summary)

This article investigates how other articles describe the roles patients, relatives and researchers have in patient and public involvement activities in health research. It also investigates which factors are supportive of creating these research partnerships. We searched four health research databases and found 70 relevant articles which somehow evaluated patient involvement activities in research. From these 70 articles we chose 40 which we closely investigated for descriptions of roles in the partnerships between researchers and patients and relatives. For this, we used a tool called the Involvement Matrix which uses five different roles: *Listener* (who is given information), *Co-thinker* (who is asked to give opinion), *Advisor* (who gives (un)solicited advice), *Partner* (who works as an equal partner) and *Decision-maker* (Who takes initiative and (final) decisions). We found that it is often researchers who take on the role of *Decision-maker* and that involvement often happens on their terms. We noticed that patients and relatives most often had the role of *partner*, when they were listed as co-authors of the article. This shows co-authorship as an authorization of their work during patient and public involvement activities. We found that patient and relative training, patients’ and relatives’ personality and communication skills, trust, financial reimbursement, and time were mentioned most often as enablers of good research partnerships.

**Supplementary Information:**

The online version contains supplementary material available at 10.1186/s40900-023-00448-z.

## Dansk lægmandssresumé

Denne artikel undersøger, hvordan andre artikler beskriver de roller patienter, pårørende og forskere har i patientinvolveringsaktiviteter i sundhedsforskning. Den undersøger også, hvilke faktorer, der virker understøttende for disse forskningspartnerskaber. Vi søgte i fire databaser for sundhedsforskning og fandt 70 artikler, der evaluerer patientinvolvering i forskning. Vi udvalgte 40 ud af de 70 artikler og undersøgte dem for, hvordan de beskrev patienternes, de pårørendes og forskernes roller i forskerpartnerskaberne. Til dette brugte vi en involverings-model, der deler mulige roller op i: *Lytter* (en der får information), *Medtænker* (en, der bliver spurgt om en holdning), *Rådgiver* (en, der giver råd), *Partner* (en, der arbejder som ligeværdig partner) og *Beslutningstager* (en, der tager initiativ og kan træffe endelige beslutninger). Vores undersøgelse viste, at det i høj grad er forskerne, der er *Beslutningstagere* i forskningspartnerskaberne og at inddragelse sker på deres foranledning. Patienter og pårørende var oftest *Partnere*, når de også var medforfattere på artiklerne og dermed bliver medforfatterskab en bekræftelse af patienternes arbejde. Vi så også efter elementer, der kan understøtte partnerskaberne og fandt at træning af patienter og pårørende, personlige- og kommunikative evner, tillid, dækning af udgifter eller betaling, samt tid var de faktorer, der oftest blev nævn som understøttende for gode samarbejder.

## Contributions by patient partners

This rapid review was conducted with a group of patients and relatives (PRs) (AKS, KB, KEB and TA), each of them are / have been a patient or carer to a patient in Denmark. Table 1 and Additional file 1: Appendix D have been developed by AKS and AWK. AKS has been part of the development of study objectives, protocol, and discussions on synthesis strategy. She has read 11 articles [1–11]; she used those to populate the tables as well as provide analytical reflections. Patient partners KB, KEB and TA read 3 Danish articles [6–8] and provided analytical reflections on these. AWK developed an open question matrix to support their work with the articles. All patient partners actively contributed to the development of the column “Patient partner observations” and brought insights to the analysis and discussion below (If the reader is interested in more information on our collaborative processes, see Additional file 1: Appendix B for a GRIPP2 short form and overview of our involvement roles using the Involvement Matrix).

**Table 1:** Overview of included articles

Author	Country	Design	Area	Cycle	Focus of article	Partner group	Framework for inclusion	Themes	Co-author-ship
Abrehart [12]	UK	Feasibility study	Pediatrics	Whole	Development of a medical imaging test for children with constipation	Children aged 8–18	GRIPP2	Relations through shared endeavor/ Learning/ Direct impact/ Consistency/ Impact on children’s development/ Building rapport	Yes
*Alexander [13]	USA	Mixed methods	Pediatrics	Whole	Childhood obesity—Evaluation of a childhood obesity treatment pilot program	Parents of obese children	CBPR	Facilitating/ Definition of success/ Training/Skills used in everyday life/ From stakeholder to PR	No
Anang [14]	Canada	Qualitative	Mental health	Whole	Suicide prevention among Inuit Youth	Youth living in suicide risk communities	CBPR	Community partnership/ Training/ Employment/ Sense of ownership/ Vulnerable group	No
*Barn [15]	Canada	Evaluation	Pulmonology	Agendas, Governance	Asthma and COPD—evaluation of a PR group as resource for several research projects	Patients with Asthma and COPD	iKT	Confidence to contribute/Training/Peer support/PEIRS-22 evaluation tool	Yes
Beeker [16]	Germany	Mixed methods	Mental health	Whole (after protocol)	Qualitative description of a process evaluations of innovative models of psychiatric care in Germany	Mental health service users	Not specified	Structural organization/ Routines/ Supervision/ Tandeming/ Heterogeneity/ Conflicts/ Power/ Personal growth	Yes
*Beighton [17]	UK	Register study	Health Services	Outcome measures, Analysis, Dissemi-nation	Data analysis of effectiveness of annual health checks for adults with intellectual disabilities	Adults with intellectual disabilities, carers	Participatory approach	Longstanding relationships/ Authenticity/ Legitimacy/ Challenges/ Dissemination/ Increased confidence	No
Birch [18]	Europe	Mixed methods	Rheumatology	Predetermined project areas	Multi-site European study on biomarkers for early detection of arthritis to predict development	Patients with arthritis and relatives	Not specified	Involvement in specific tasks/ Impact on study process/ Training/ Feedback/ Impact on dissemination/ Inability to contribute	Yes
*Bourque [19]	Canada	Mixed methods	Oncology	Whole	Survivor needs of adolescent and young adults with brain tumors	Parents of—and youth with prior brain tumors	The Nesting Dolls Design	‘Sherpas’ as stakeholders/ Empowerment/ Inclusion in whole process/ Capability to participate and facilitation of this/ Adapting and sharing knowledge	Yes
Brutt [20]	Germany	Review	Mental health	Outcomes, Analysis, Lay Summary	PPIE in a systematic review on metacognitive interventions	Mental health service users	Not specified	Focus group discussions/ Predefined protocol/ Remuneration	No
*Burrows [21]	UK	Mixed methods	Digital health	Design	Evaluation of collaboration in a large digital development study on home health technology	Not specified/ Advisory group	Gradinger et al.’s value systems/ GRIPP2	Advisory groups/ Systematic approach to involvement/ Value systems	No
*Carr [22]	Canada	Survey	Rheuma-tology	Whole (after topic selection)	Co-design of patient experience survey	Patients with arthritis	PED framework	Peer-to peer with PaCER facilitators/ Online meetings/ Recommendations to research group	Yes
Castensøe-Seidenfaden [23]	Denmark	Mixed methods	Digital health	Whole	Development of app for self-management of type 1 diabetes mellitus	Young people with type 1 diabetes	Participatory Design	Conflict solving/ Value of diverse teams/ Separate steering group and PRs	No
*Chiu [24]	USA	Mixed methods	Oncology	Whole	Psychosocial impact of participating in activities (dragon boating) for breast cancer survivors	Breast cancer survivors	Participatory research	Development of survey/ Power sharing/ Mutual benefit/ Reciprocity	No
*Cook [25]	UK	Service design	Mental health	Whole (after topic selection)	Creating a Mindfulness-based course to support parent carers	Parent carers for adults with learning disabilities	Action Research/ Participatory Health research	Communicative spaces/ Impact: researchers, project	No
*Dawson [26]	UK	Qualitative	Minority health	Whole	PPIE in a doctoral project on inclusion of minority groups in PPI	BAME	INVOLVE	Relationship development/ PR reflections/ Researcher reflections/ Mapping research values	Yes
Dennehy [27]	Ireland	Qualitative	Digital health	Whole	Evaluation of the work an advisory group of young people did in a qualitative research project	Young people at risk of cyber bullying	Lundy’s Model of Participation	Voice/ Influence/ Right to be included	No
*Devonport [28]	UK	Intervention	Obesity and binge eating	Design, Analysis	Reflections on an intervention study on emotional eating with patients and practitioners	Patients with binge eating disorders/ weight management	INVOLVE	Group dynamics/ Development of relationships/ Difference in knowledge and what is shared/ Rights-based approach	No
Dewa [29]	UK	Qualitative	Mental health	Whole	Reflections on co-production of a qualitative interview study	7 Young people with mental health difficulties	McPin Foundation Priority Setting/ James Lind Alliance	Describing the study against principles of coproduction/ Recommendations/ Coproducing interviews and analysis	Yes
*de Wit [30]	Nether-lands	Evaluation	Rheuma-tology	Predeter-mined project areas	Evaluation of a pilot study model for structural involvement in rheumatology research	Patients with arthritis	FIRST model	Challenges/ Guidance/ Researcher and patient needs/ structural partnerships	No
Dovey-Pearce [31]	UK	Service design	Health Services	Unclear	Reflections on a 5-year longitudinal health research program supporting young people transition to adulthood	People with intellectual disabilities and carers	INVOLVE/ GRIPP	Longstanding relationships/ Learning outcomes	Yes
*Faulkner [32]	UK	Qualitative	Mental health	Whole	Building capacity to support mental health service users’ experiences of hate crimes	Survivors of hate crime due to disabilities	Survivor research/ INVOLVE	Description of methods and process to demonstrate value of the approach/ User-led research/ Legitimacy/ Program development/	Yes
*Frankena [33]	Europe	Case study	Several areas	Whole over several projects	Evaluation of 4 European case studies in health research including people with intellectual disabilities	People with intellectual disabilities	Rohlfing’s integrative framework	Partnership development/ Outcomes for all partners and projects/ Legitimacy	Yes
*Froggatt [34]	UK	Mixed methods	Health services	Design, Delivery	Qualitative evaluation of PR involvement in an evaluation on health promotion in primary care	Care home residents	APPROACH	Benefits/ Relationships/ PR management	No
*Gammon [35]	Norway	Service design	Mental health	Idea, Design	Case study evaluation of service user involvement in the design phase of an online tool for self help	People with mental illness	CBPR	Benefits for study and participants/ Partnerships/ Mutual learning/ Legitimacy	Yes
Grant [36]	UK	HTA	Digital health	Design	Developing E-mental health platforms for school children	Young school children	Not specified	Impact: project/ Focus groups	No
Grundry [37]	UK	Qualitative	Mental health	Whole	PRO-measures on quality of life in mental health service users	Mental health service users	Not specified	Impact/ Evaluation	Yes
Gupta [38]	UK	Case study	Mental health	Dissemination	Case study of the development of dissemination materials on mental health research to the public	Mental health service users	Not specified	User-researcher interactions/ Benefits/ Challenges/ Dissemination/ Empowerment/ Low- and middle-income countries	No
*Hitchen [39]	UK	Service design	Mental health	Whole (after topic selection)	Integrating user and carer views on implementation of self-directed support	Mental health service users, carers	Action Research	Professional talk/ Trust/ Legitimacy/ Empowerment/ Benefits/ Shared learning/ Safe spaces/ Remuneration	No
*Hoekstra [40]	USA	Qualitative	Neurology	Design, Analysis, Dissemination	Qualitative investigation of patients and researchers’ experiences participating in spinal cord research	Spinal cord injury patients	iKT	Dissemination/ Building knowledge together/ Researcher and participant reasons to PPIE/ Building relationships/ Valuing perspectives/ Role models	Yes
*Honey [41]	Australia	Evaluation	Mental health	Whole	Evaluation of a consumer-led evaluation of a mental health program	Prior mental health patients	Collaborative auto-ethnography	Reflexivity and its impact on partnerships/ Academics with history of mental illness	Yes
*Hutchinson [42]	UK	Qualitative	Mental health	Whole	Qualitative research on mental health users’ experiences being co-researchers in an IPA study	Mental health service users	PAR	Empowerment/ Transformation/ Impact: personal/ Reframing a narrative/ User-researchers	No
*Jewell [43]	UK	Register study	Mental health	Predete-mined project areas	Evaluation of a service user and carer advisory group for mental health data linkage research	Mental service users	GRIPP	Advisory boards as point of contact for researchers/ Researcher behavior/ Impact: projects, researchers/ Training	No
*Jørgensen [6]	Denmark	Service design	Oncology	Whole	Evaluation of PPIE in a project focusing on empowerment and development of PRO.measures	Patients with cancer	GRIPP2	Discussion of methods and practice/ Views and experiences of researchers and patients from the study/ Type of patients involved/ Local context	No
Kara [44]	UK	Review	Mental health	Whole	Coproduced literature search and evaluation of a mental health carers research reference group	Carers of mental health service users	Participatory evaluation	PR-lead research/ Advisory groups in research/ Impact: projects/ Organizational space for advisory groups/ Benefits	No
*Kearns [45]	UK	Survey	Neurology	Design	Development of a questionnaire for future use in aphasia rehabilitation	People with aphasia	INVOVLE	Group dynamics/ Experiences/ Development of discussions/ Goals/ Researcher self-reflection	No
Lammons [46]	UK	RCT	Pediatrics	Design	Evaluation of PPI in first phase of an RCT study on preterm nutritional care	Former neonatal intensive care patients and parents	Not specified	PR view on RCT process/ Legitimacy from emotions/ Researcher hesitations	No
Leese [47]	Canada	Qualitative	Rheumatology	Whole over several projects	Co-produced study on PRs experience of PR-researcher relationships in health research	Patients with arthritis	Not specified	Patient experience/ Being heard/ Legitimacy/ Co-building social relations/ Hard work for both parties/ Ethics/ PR-led research	Yes
Liabo [48]	UK	Qualitative	Various	Whole over several projects	Qualitative co-produced self-evaluation of three public involvement health research groups	Patients, carers, members of public involvement groups	Not specified	Values in practice/ Principles of involvement in practice/ Challenges	Yes
Lincoln [49]	USA	Qualitative	Mental health	Whole	Qualitative interview study of needs when transitioning from child to adulthood with mental health issues	Young adults with a history of mental illness	CBPR	Training/ Relationships/ Impact: project/ Remuneration/ Giving voice to underrepresented groups	No
*Lindblom [11]	Sweden	Qualitative	Rehabilitation	Design	Co-design of person-centered transition from hospital to home	Patients with stroke and relatives	Arnstein’s ladder/Human-centered approach	Roles and power/ Shared understanding/ Participation via interaction/ Flexibility/ Types of researchers	No
Locock [50]	UK	Qualitative	Mental health	Analysis	Evaluation of PR involvement in analysis of qualitative interview study on quality improvement	Mental health service users and stroke patients	Not specified	Empowerment/ Impact: researchers, PRs, project/ Training	Yes
*Marks [3]	UK	Mixed methods	Nephrology	Whole (after topic selection)	Personal reflection on participation in a patient experience improvement study in the renal field	A patient with a renal condition	GRIPP2/ INVOLVE	Difference between advisory group and co-researcher/ Reflections on different stages of research cycle/ Role as co-researcher	Yes
*Melchior [51]	Nether-lands	Qualitative	Palliative care	Whole	Qualitative study on PPIE processes in 10 studies	Patients, caregivers not further specified	PAR	Participation cultures: Relationship, task and control/ Impacts of culture/ Relationships	Not clear
*Miah [1]	Europe	Mixed methods	Dementia Research	Whole	Qualitative evaluation of PPIE impact in a multi-site dementia research program	People with dementia and their carers	GRIPP 2	Impact: project, PRs, researchers, personal/ Training/ Resources/ Multi-national project	No
Minouge [2]	UK	Service design	Health Services	Whole	Development of a training package for PPIE in health research	Not specified	Not specified	PR-led research/ Experiential knowledge/ Partnership dynamics/ Structural changes	No
Mjøsund [52]	Norway	Qualitative	Mental health	Whole	Description and evaluation of analysis methodology in a mental health promotion project	Mental health service users or their carers	Not specified	PR improves quality of research/ Role of PR-team/ Using interpretive phenomenological analysis/ Power of multiple perspectives/ Skills	Yes
Mockford [5]	UK	Qualitative	Dementia Research	Whole	Development of service user-led recommendations around discharge from acute care to community care	Patients with Alzheimer	Not specified	Structural changes/ Organizational culture/ Training/ Remuneration/ Motivation	Yes
*Nichols [53]	UK	Feasibility study	Neurology	Whole	Chronic headache management—Evaluation of a self-management program development	Patients with chronic headaches	GRIPP2	PPIE in whole process/ Equality/ Using skills for right tasks/ Roles/ Rules of engagement/ Building relationship/ Remuneration	No
*Nierse [10]	Nether-lands	Agenda setting	Nephrology	Agenda	Research agenda setting with patients in a patient organization	Patients with chronic kidney disease	Responsive methodology	Group dynamics/ Dialogue/ Researchers as facilitators/ Empowerment / Building bridges between science and society	Yes
*Nissen [9]	Denmark	Feasibility study	Oncology	Predetermined project areas	Cancer rehabilitation—a feasibility study of a psychosocial mindfulness intervention	Patients with breast and prostate cancer	INVOLVE	Impact on project/Structural organization of PPIE / Challenges	No
Noyes [54]	UK	Service design	Health Services	Whole	Report co-productive strategies for a qualitative evaluation of a new soft opt-out system on organ donation	Family members of deceased donors	Not specified	Outcomes: preset measures/ PPIE as response to methodological challenges/ Vulnerable groups	Yes
*Nöstlinger [55]	Nether-lands	Prevalence study	Epidemiology	Whole	Epidemiological study testing the prevalence of HIV amongst immigrants	People living with HIV	CBPR	Partnerships/Team leadership/PR training as researchers/Ways of contributing	No
Olding [56]	Canada	Survey	Substance abuse	Design	Co-development of a patient-reported experience questionnaire for people who use drugs	Drug users	Not specified	Graphic facilitation/ Identifying unmet needs/ Ethical considerations/ Remuneration	No
*Ostrach [57]	USA	Qualitative	Health services	Design	Evaluation of the process of the development outpatient women’s health screening tools	At risk women	Human-Centered Design	Timing and trust/ Historical mistrust/ Relationships/PR perspective	No
*Pallesen [58]	Ireland	Intervention	Health Services	Design	Stakeholder evaluation of co-designing a leadership intervention to health care teams	Not specified	Experience based Co-design	Sharing experiences/ Legitimacy/ Relationships/ Feedback/ Sharing power/ Storytelling	No
Pinfold [59]	UK	Service design	Mental health	Proposal, Analysis, Dissemi-nation	Evaluation of a study on personalization in mental health policy	People with mental health problems	Not specified	Team building/ Lack of remuneration and training had impact on involvement level/ Inequalities/ Lived vs research experience	Yes
*Pomey [4]	Canada	HTA	Cardiology	Design	Evaluation of a project creating recommendations for cardiac defibrillator replacement	Patients with cardiac defibrillators	Own	Value of PPIE/ PPIE in literature review/ Co-construction of results/ Impact: researchers and project/ Challenges	Yes
*Rayment [60]	UK	RCT	Pediatrics	Design	Pilot trial examining the effects of probiotics during pregnancy and risk of preterm birth	Mothers from populations at risk of preterm birth	Nominal group technique for discussion groups	Discussion groups/ Advisory team throughout the RCT/ Impact: project/ Process tailored to the needs of PRs	No
Ruff [61]	USA	Survey	Health Services	Design, Implemen-tation	Designing and implementing a survey on mental needs of children in foster care transitioning into adulthood	Young adults with a background on foster care	CBPR	Building relationships/ Vulnerable groups/ PR consultants as bridge to target group/ Limitations/ Agency	No
*Seeralan [62]	Germany	RCT	Mental health	Design	Development of patient-targeted feedback intervention in primary practice	Patients with experience of depression	INVOLVE	PR-led workshops/ Importance of structure and researcher skills/ Impact: project/ Remuneration	No
*Sharmil [63]	Australia	Qualitative	Minority health	Whole	Aboriginal health research related to consumption of drugs and alcohol to improve health service delivery	Aboriginals at risk of substance abuse	PAR combined with aboriginal traditions	Researcher and PR joined forces/ Researcher learnings from subject population/ Adjusting research process to subject needs/ Incorporate PR knowledge	Yes
*Simpson [64]	UK	HTA	Health Services	Whole	Creation of an early awareness and alert system and related webpage	Not specified	INVOLVE	MedTech involvement/ Impact: product/ Various methods in one project/ Social Media/ Challenges/ Knowledge sharing	No
*Skovlund [8]	Denmark	Intervention	Oncology	Whole	Clinical controlled intervention trial on effects of using PRO before metastatic melanoma consultations	Patients with or with history of melanoma	INVOLVE	Focus on analysis/ Training/ Structural and emotional challenges/ Remuneration/ Building relationships/ Skills	Yes
Springs [65]	USA	Review	Health services	Whole	Evidence synthesis on integrating arts-based interventions in health care	Various patients, artists	PCORI	Dissemination/ Training helps legitimacy/ Confidence	Yes
Stocker [66]	UK	Qualitative	Health services	Analysis	Critical reflection on collaborative data analysis in a care home-study	Relatives of care home residents	Not specified	Multiple professions/ Role play/ Steering choices/ Lack of practical guidance/ PR Interest group	No
Thomas [67]	UK	Qualitative	Mental health	Whole	Mental health research—a reflection on a study exploring deprivation as a trigger for mental stress	People at risk of developing mental health issues	Not specified	Lack of equality/ Importance of socializing/ Power dynamics/ Trust/ Relational work/ Underserved communities	Yes
Tremblay [68]	Canada	Qualitative	Minority health	Whole	Evaluation of developing a design for including indigenous patients in research partnerships	Indigenous Canadian tribes	Not specified	Training/ PPI as validation of results/ Recruitment/ Trusting relationships/ Capacity building/ Historical context	Yes
*Vat [69]	Canada	Evaluation	Several areas	Whole, several projects	Co-designing an evaluation of 11 projects with PPIE	Not specified	Several local patient and public evaluation tool kit	Human resources needed from both parties/ Tokenism/ Learning as legitimacy/ Integrating experiential knowledge	Yes
*Vogsen [7]	Denmark	Clinical trial	Oncology	Design, Analysis, Dissemi-nation	Evaluation of PPIE impact on retention and recruitment in a clinical trial	Women with prior breast cancer	GRIPP2	Researcher hesitation/ Involvement in Patient-related activities/Involvement increased over time/Dissemination impact in community	Yes
*Worsley [70]	UK	Health services	Mental health	Proposal	Development of a public-led research proposal on improving quality of therapeutic relations	Users of mental health services	James Lind Alliance	Writing grant applications/ Validating lived experience/ Remuneration/ Equality and respect/ Marginalization within the group/ Structural issues	Yes

BAME = Black, Asian and Minority Ethnics, RCT = Randomized Controlled Trial, HTA = Health Technology Assessment, PPIE Patient and Public Involvement and Engagement, GRIPP2 = Guidance for Reporting Involvement of Patients and the public, PRO = Patient Reported Outcomes, PR = Patient or Relative, CBPR = Community-based Participatory Research, COPD = Chronic Obstructive Pulmonary Disease, PAR = Participatory Action Research, iKT = integrated Knowledge Translation, PCORI = Patient-Centered Outcomes Research Institute. * = Part of narrative analysis in Involvement Matrix (by reference number) and metasynthesis. Note: Barn 2021 and Nichols 2021 are not represented in Matrix, as these articles did not describe roles

## Background

In the last decades, involving patients in developing and conducting health research projects has become a way of achieving high quality and efficient integration of health care [71] as well as improving the overall quality of health research [72]. When PRs are involved not as research subjects but as research partners in the health research process, it can lead to: “*meaningful change in patient outcomes and health systems, and realigning both research processes and outcomes to be patient-centered*” [73]. PR involvement has become a demand for many funding programmes and journals as well as a health policy prioritisation [74]. Despite increasing numbers of projects involving PRs, studies have highlighted unclear definitions of *involvement* and describe tensions when trying to validate experience-based knowledge in the medical field [75].

Many different labels exist to describe involvement processes: co-production, co-design, collaboration, involvement, engagement, patient and public involvement, community based participatory research, participatory action research and others in research reports. These concepts and methods are rooted in traditions of different research disciplines and contexts [76], but whether the involvement method or label used to describe this, has implications on the partnerships and outcomes is unclear [77]. Recent literature has looked at partnership practices and found that: role definition and partner expectations are necessary prerequisites for the partnership to be successful. Respect, equitable power, trust, transparency, shared and collaborative decision making has been described as foundational principles for research partnerships; and patients taking on different roles during research partnerships, such as: members of research teams, advisory groups, steering committees and working groups, consultation, and specific research tasks [73, 74, 78]. These authors recommended further detailed analysis of partnership role characteristics and what impact they have. Others concluded that we lack knowledge on how impact and outcomes are achieved in these collaborative partnerships [79] and how these partnerships might be similar or different [80]. It should be noted that the role of the researchers in research partnerships has been investigated much less than the role of patients [81].

## Objectives of this study

We studied peer-reviewed articles describing PPIE activities for the roles researchers and PRs fulfil in different types of involvement activities, the factors that enable involvement, and how PRs’ knowledge is utilised. As part of that:

- How do patients and relatives establish themselves as knowledgeable?
- What roles do researchers, patients, and relatives have in enabling partnerships?

The term Patient and Public Involvement/Engagement (PPIE), which has previously been described as internationally representative to cover the wide range of involvement activities and methods [76], will be used in this article.

## Methods

This study used a rapid review approach to obtain a systematic overview of articles describing PPIE in health research followed by a narrative analysis and meta synthesis of selected articles. Rapid reviews are a newer form of review and are described as an: “*assessment of what is already known about a policy or practice issue, by using systematic review methods to search and critically appraise existing research*” [82]. By omitting or simplifying the systematic review process, it has the benefit of providing an overview without requiring substantial resources and time and thereby helps closing the gap between decision making and evidence generation [83]. It has an explorative character and is suited to investigate new trends as it gives an overall quality or direction of the literature available [82].

Our methods were inspired by Haby [83], Dobbins [84] and Boden et al. [85]. As there is no set way of conducting a rapid review, we highlight the steps altered from a systematic review:

- development of a high specificity search string,
- limitation of databases to four,
- no use of grey literature,
- time limit on publications,
- main screening performed by the first author,
- no systematic quality assessment,
- in-depth analysis limited to a selected group of included articles.

**Fig. 1:**
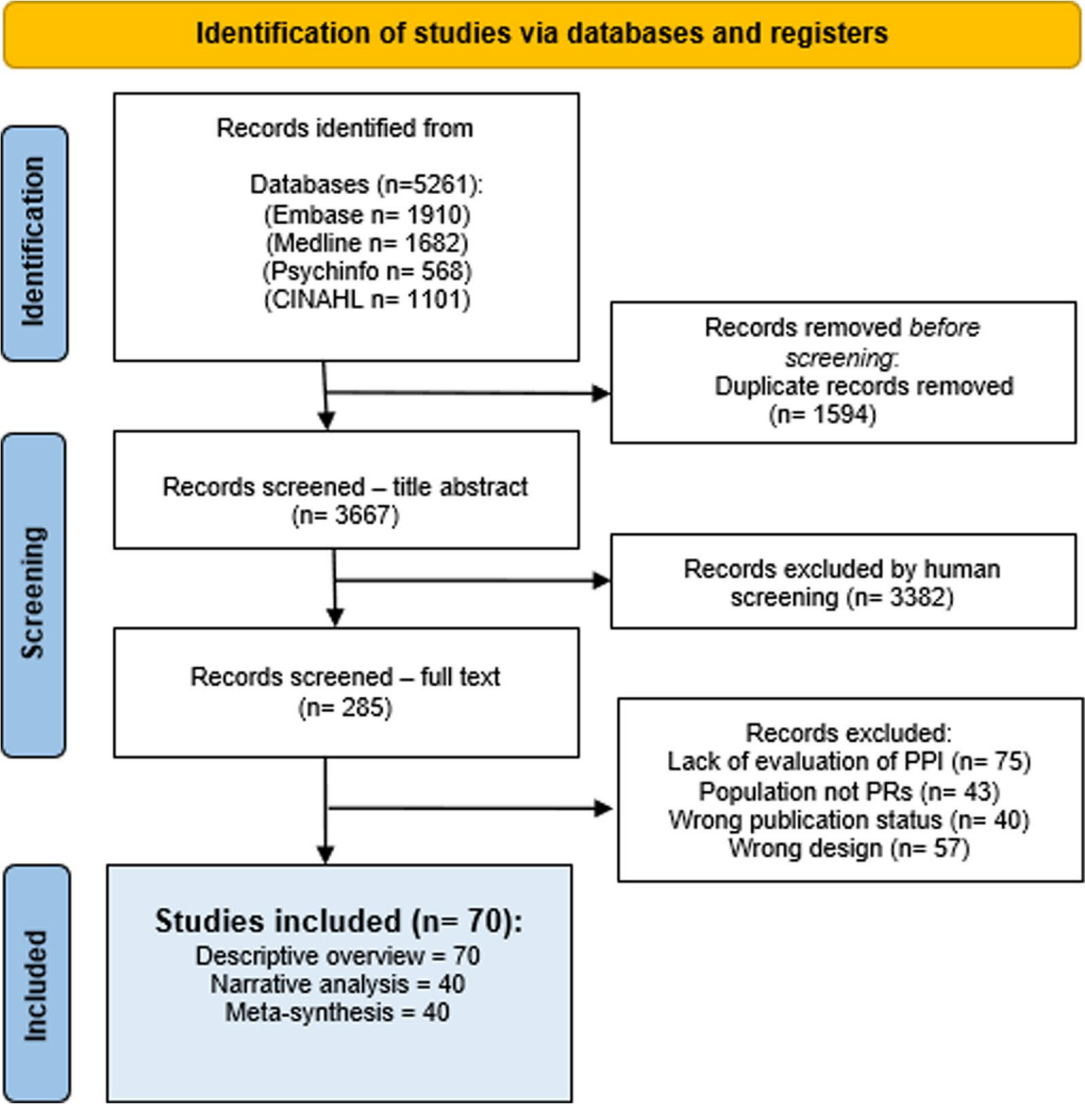
PRISMA Flowchart

See Additional file 1: Appendix C for a justification of each of these choices. The selection process of included papers follows the PRISMA guidelines [86] (See Fig. 1), and the reporting uses the PRISMA 2020 checklist [86] where items are applicable according to the used rapid review methodology.

In accordance with this study’s overarching principles of accessibility, transparency and reciprocity as basis for research partnerships, the protocol for this rapid review was co-developed and registered with the Open Science Framework on Nov 25th 2021 [87] https://doi.org/10.17605/OSF.IO/QMWVK). Alterations to the protocol can be found in Additional file 1: Appendix C; main changes included only performing narrative and meta analysis on a selection of included article.

### Eligibility criteria

Inclusion criteria were: Published articles presenting practical examples and reflections, case studies, interviews, ethnographies, or evaluations of research partnerships between PRs and researchers in any type of qualitative or quantitative studies (see Additional file 1: Appendix C for detailed inclusion and exclusion criteria). The Guidance for Reporting Involvement of Patients and Public (GRIPP) guidelines, a checklist for reporting PPIE activities in health research first published in 2012 [88] (and the in 2017 revised GRIPP 2 [89]) first offered a comparable framework to describe and report involvement practices. Therefore, we excluded papers published prior to 2012. The population was defined as patients of any age with any (past or present) medical condition, relatives or caregivers participating as stakeholders, panellists, co-designers etc. at any point of the research cycle.

### Search strategy

Searches were run in four databases (Medline, Embase, PsycInfo and CINAHL) in November 2021 and rerun in February 2022. It has been noted previously how reporting on patient involvement activities varies: sometimes not mentioned in the title or abstract [90] and challenging to capture in (standard) search terms [80]. Therefore, we used the quality-tested patient involvement search string developed by Rogers et al. [90] and Cooke & Smiths’ SPIDER-tool [91] incorporating search blocks on study design and research type for higher specificity. The search string was developed for Medline and translated with the assistance of a research librarian to match the other databases. See Additional file 1: Appendix A for SPIDER-tool (Additional file 1: Table S1) and Medline search string (Additional file 1: Fig. S1).

### Data analysis

To provide an overview of study characteristics of included articles as well as an in-depth analysis of roles, enablers and outcomes, the results were synthesized as follows:

- Overview of study characteristics of all eligible papers reporting on PR/researcher partnerships using a matrix to extract descriptive information.
- Narrative analysis of partnership roles of selected articles using the Involvement Matrix.
- Meta-synthesis of PPIE enablers of selected articles.

*Study characteristics* We conducted a descriptive summary of all 70 papers extracting data on geographical origin of project, research area and design, format of PR group, focus of article, philosophy for inclusion, themes discussed and PR co-authorship. This information was extracted from all parts of the papers; a thorough read and reread for each paper was required. Our goal was to create a searchable overview of relevant practical PPIE examples readily available for interested readers (Table 1). As such, we aimed to deliver towards one of the rapid review’s functions of providing clarity and accessibility of research evidence [83].

*Narrative analysis* As we found more eligible papers than anticipated we discussed how best to give an overview of available evidence in the timeframe available. We selected 40 papers which we considered most comparable (depicted with an asterisk in Table 1), according to the following rationale:

- partnerships with adult patients and relatives (as research indicates that extra steps have to be taken to enable participation of children and youth [92]),
- articles that reported the framework used to account for and or support their involvement activities either in the background or methods section (as we aimed to investigate different types of partnerships, we found it useful to understand the framework behind the PPIE activities),
- Excluding systematic reviews (as detailed Cochrane guidelines on PR involvement in systematic reviews are available [93] and we considered this a readily available aid for researchers).

**Fig. 2:**
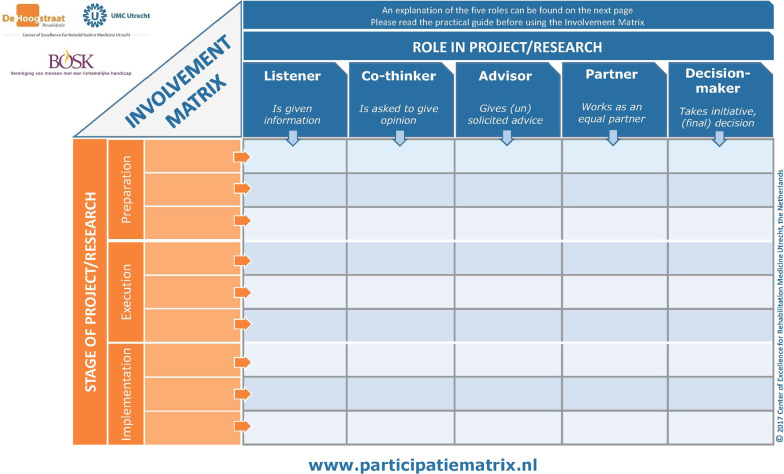
Involvement Matrix (reprinted with permission)

We used Smits and colleagues’ Involvement Matrix to perform the narrative analysis (see Fig. 2). The matrix was designed as a conversation-tool to discuss roles and expectations to support PPIE in research [94]. We have used the matrix to also describe researchers’ roles, as to our knowledge no model for analysis of researcher roles in partnerships with patients exists. We scrutinized the full papers for descriptions of roles to populate the matrix. This information was scattered throughout the papers; sometimes it was found in the methods section, but most often in designated PPIE-headlined sections or in the contributions or acknowledgement section. Authorship requires substantial contributions to the research process and article write up as recommended by the International Committee of Medical Journal Editors [95]; therefore, to understand different levels of partnership, we stratified the 40 articles in PR co-authored papers (n = 16) and non-PR co-authorship (n = 24). All authors discussed the extracted data.

*Meta-synthesis* Finally, a meta-synthesis of enablers and outcomes described in the 40 articles was performed. Papers were analysed for value creation and outcomes related to and enabling actions in the partnerships. This information was found under findings, evaluations, self-reflections, or discussion sections depending on the scope of the article. Those sections were thoroughly read and information was extracted using a purposefully-developed matrix. The information was then synthesised and reported in Fig. 5.

## Results

A total of 5261 potential hits were collected in Endnote 20 2.01 and transferred to Covidence 2.0 (Covidence.org) for screening and full text analysis. After deduplication, 3667 records were screened for title and abstract. Twenty-five percent of the records (918) were independently screened by two researchers (AWK and MLK) and inclusions compared for disagreement (15%). Disagreements were discussed and resolved by an external referee. The remainder of articles were screened by one researcher (AWK), who also screened the 285 articles eligible for full text reading. Seventy articles were included for analysis. A searchable full list is available at the Open Science Framework and will be available on the website for Center for Research with Patients and Relatives at Odense University Hospital [96].

### Descriptive overview

**Fig. 3:**
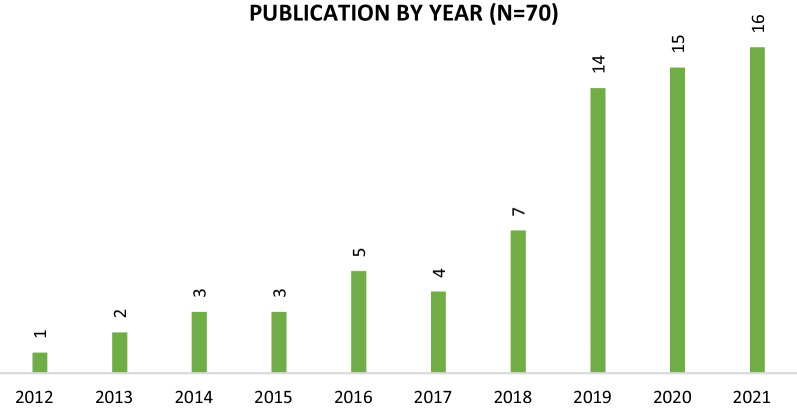
Articles grouped per publication year

We found 70 articles eligible for inclusion; the total number of articles reflecting on and evaluating PPIE activities increased progressively between 2012 and 2021 (see Fig. 3).

The geographic origin of studies is polarized predominantly between the UK (n = 32), Canada (n = 9), and the USA (n = 7). Other industrialized countries account for minor contributions: Denmark (n = 5), Netherlands (n = 4), Germany and European collaborations (n = 3), Norway, Ireland and Australia (n = 2) and Sweden (n = 1). Different research areas are represented (see Table 1) with mental health leading with 21 publications, followed by health services research (n = 11), and oncology (n = 6). A qualitative research design was most frequently used (n = 21), but more traditional biomedical research designs such as clinical trials, randomized controlled trials (RCTs), health technology assessments (HTA), register studies, surveys and reviews were also represented (see Additional file 1: Appendix D Table S2 for visualization of results). The included articles cover a wide range of involvement formats from advisory boards, being consulted once on a project [97], to full coproduction at all stages of a project [1]. All steps in the research cycle are represented: from research agenda generation to dissemination activities. Thirty-eight of 70 papers reported including patients in the whole research cycle; if patients were involved in just one step, the design phase (n = 11) was most frequently reported. Using authorship listing and information in the contribution and affiliation sections, 33 articles were identified as being co-authored by PRs. For one article we were unable to determine whether PRs had been co-authors, and thus classified this [51] as non-co-authored. Articles described inviting PRs with lived experience of the condition or service under investigation, yet in 5 articles the authors did not report any details on who the PRs were or reasons for selecting them [2, 21, 58, 64, 69].

Articles mentioned a total of 21 different frameworks for involving PRs, yet a large group of articles (n = 19) did not mention a specific framework guiding the collaborative processes. Geographical differences in the frameworks used can be seen with only European and UK-based articles referring to INVOLVE guidelines and/or the GRIPP reporting tool—both originating from the UK. In articles with no frameworks specified, 12 out of 19 had PRs listed as co-authors.

Authors described both positive experiences and challenges related to PPIE activities. However, all 70 articles report that the involvement activities ultimately resulted in positive changes in the projects, ranging from researchers gaining new perspectives on their project [12], reformulation of questions in questionnaires [37], changing the intervention design [35], and collaboratively developing guidelines [4]. A few articles mentioned how the researchers were worried that the PPIE in their research project would decrease scientific rigour [69], not be taken seriously [32] or fail to obtain legitimacy amongst clinicians [35].

### Narrative analysis of partnership roles

**Fig. 4:**
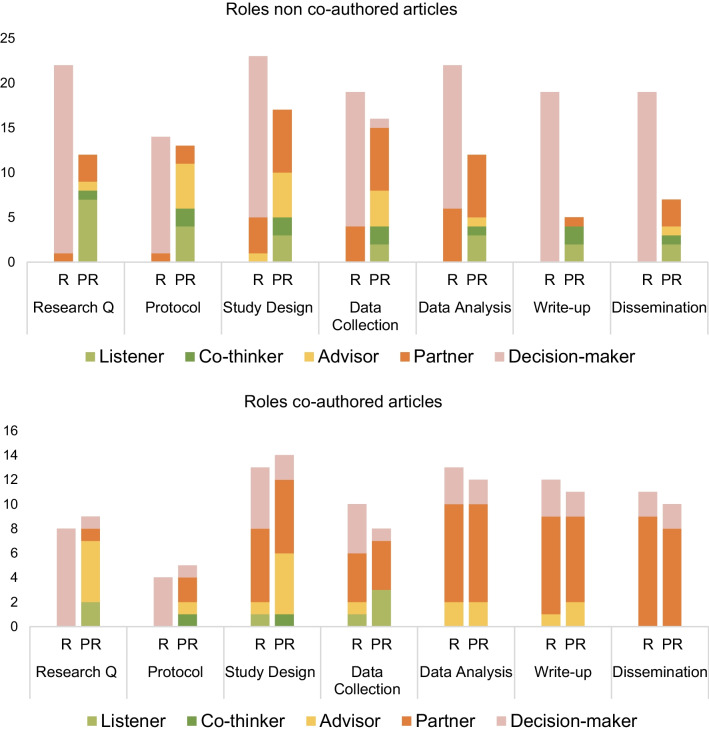
Visualization of roles within non-co-authored and PR co-authored articles using the Involvement Matrix. Research Q = Research Question, R = Researcher, PR = Patient & Relative

The role of both researchers and PRs in the partnerships was determined using Smits et al., Involvement Matrix in the 40 selected articles marked with an asterisk in Table 1. The results show that PR roles in non-co-authored articles are: listener, co-thinker, advisor, and partner. One article mentioned that PRs had decision-making authority [42]. See Fig. 4 and (Additional file 1: Table S3 in appendix for data details). PRs’ roles changed during the projects throughout the research cycle: In the earlier stages of research question and protocol development, PRs’ role is most often described as listener or advisor. This was difficult to determine as few articles reported clearly on PRs' roles in the early stages of the research cycle. During the design stage PRs most often had the role of *advisor* or *partner*. The role of *partner* was often described where PRs had been involved in the design, data collection and analysis stage. When PRs were involved in several steps of the research cycle they were more frequently described as partners in the project. Few articles described PRs being involved in the write-up and dissemination stage [6, 24, 42]. The most common role for the researchers was ‘Decision-maker’ (the one who takes initiative and/or makes final decisions). This role did not change during the research cycle.

PR role in PR co-authored articles was most frequently described as partner—especially at the execution and implementation stages. In the early stages of the research cycle, PRs had the role of advisor or listener. Again, few papers described the early stages of the projects clearly, therefore information is missing, and results should be treated with caution. After research question development, researcher role in PR co-authored articles was also most frequently described as partner. Their role seemed more flexible and change as the project progressed through the research cycle and several of the projects described PR and researcher roles as dynamic and shifting between both parties taking the lead and partnering.

### Meta-synthesis of partnership enablers and outcomes

**Fig. 5:**
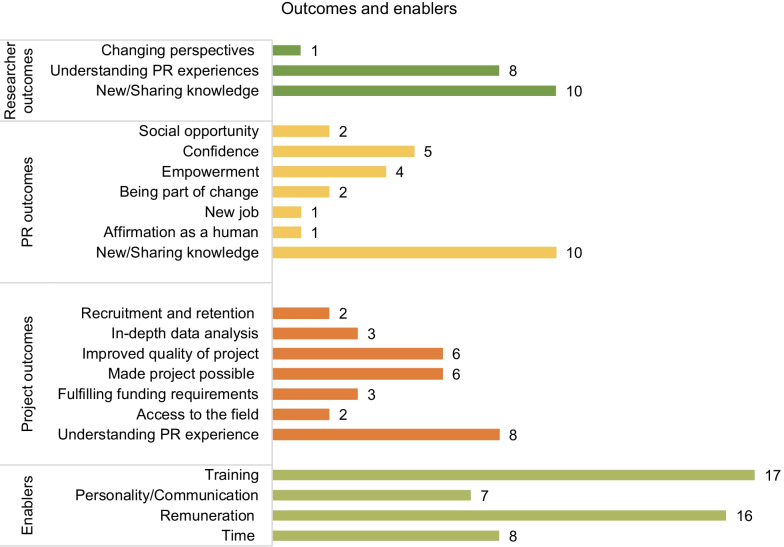
Overview of enablers and outcomes for PR partnerships in included articles (n = 40)

Included articles described several enablers that make the partnership or make it possible. An overview is presented in Fig. 5.

*Training* Several articles reported that researchers had provided training for the PRs to be able to contribute to the projects [1, 6, 13, 15, 22, 26, 34, 35, 42, 43, 55, 62, 69]. PRs also mentioned that they needed training and support to gain confidence to contribute at the same level as the researchers [1, 13, 15, 30, 43, 58]. A few articles [15, 30, 34, 69] mentioned training for researchers in PPIE as it was a novel territory for them, and one article [63] described that the PRs taught the researcher in their understanding of health and disease.

*Personality and communication skills* Finding a common language stripped from medical jargon was described as important; one paper mentioned that PRs made researchers aware when this happened [35]. PR personality and communication skills were described as important in the partnerships: being “pleasant collaborators” [9], to be “able to reflect their experience in a wider context” [4], “having the physiological and psychological means to contribute” [7], having “interpersonal skills to facilitate collaboration” [4], being able to “expresses him/herself clearly and simply” [4], and one patient mentioned “communication is my key skill” [3]. Two articles mentioned that researchers can also have strong personalities [11, 58] which can hindrance to the partnership.

*Remuneration* Six out of 24 non-co-authored articles [9, 24, 39, 42, 55, 70], and 9 out of 16 co-authored articles [3, 4, 7, 8, 15, 26, 32, 35, 41] mentioned remuneration or travel reimbursement as important. A few PRs declined [4, 6, 9] explaining it would change the relationship and bring more responsibility, or accepted remuneration, but felt as a volunteer [3].

*Time* The more steps the PRs are involved in, the more the influence of the PRs on the project and partnerships was reported as strong [9, 39, 51, 53] suggesting a longitudinal causality. Time was also mentioned as a factor in shorter partnerships as time spent on informal talks pre- and post-meeting helped build the relationship [15] and allowed for time spent together to reflect during the process [41]. However, time could also be a challenge, for example due to time pressure of other competing research activities [62]. Activities were less successful when “substantial” time and efforts were needed to organize and plan meetings [51] and when PRs had to spend time on activities in between meetings [9]. It was also mentioned that moving beyond consultation with PRs required extra time and workload [8].

*Trust* When PRs felt that they “were actually listened to” [17, 32] it helped the collaboration and created trust between the parties. Some PR co-authored articles described as PPIE intrinsic to the research project as PRs’ knowledge and perspectives actually made the research possible [19, 26, 32, 35, 40, 41, 55, 63]. In these papers PRs were described as: partners to the researchers in facilitating and conducting interviews [19], shared decision-makers when “working alongside researchers to coproduce interpretations” [32], essential to the research because they were gate-keepers to the community, a critical friend and [26, 55], the ones who accepted the researcher [63] and validated and consolidated the researchers’ point of view [35]. The researchers were trying to obtain legitimacy and gain PRs’ trust by initiating partnerships on the PRs’ terms. The researchers did so by focusing on creating supportive environments and levelling out power differences and by actively seeking acceptance of their suggestions.

### The outcomes of PPIE

The outcomes reported for both co-authored and non-co-authored articles focus on impact for the research project (see Fig. 5). Authors mentioned: increased recruitment and retention in the studies as a result of the PPIE [7, 34], more in-depth data analysis [6, 39, 42], improved quality of the project [6, 11, 17, 33, 45, 58, 98], creation of new knowledge and exchange of knowledge of and aspects of a disease [1, 25, 28, 30, 42, 53, 55, 57, 62, 64], fulfilling funding requirements [6, 9, 43], and gaining access to the field of study [26, 55]. Personal accounts of PRs were persuasive [35] and changed researchers’ perspectives [19]. A few articles mentioned that the goal of collaborating with PRs was to create the research project around the PRs’ experiences. The PR groups here included black, Asian and minority ethnics [26], aboriginals [63], abuse survivors [32], mental health services users [39, 42, 70], people with spinal cord Injuries [40], and people with aphasia [45]. The value of working with PRs in those partnerships was described as “to enable service users to find voice and freedom” [32], to create research which makes a change in the lives of people [40], a way of bringing disadvantaged groups into research [63]. Articles reporting on personal value for PRs mentioned benefits as: a social opportunity to meet fellow experiencers [15, 45], to gain confidence [17, 28, 30, 33, 55] and feel empowered [17, 28, 39, 55], to be part of change or improvement [8, 33], to find a new job [39], and to get affirmation as a human being [42].

## This project’s patient and relative partners’ observations and reflections

We, the patient and relative panel, selected 11 of the 70 articles to read and analyse. We selected Danish articles (n = 5) because we are from Denmark and sought to find a certain familiarity with the research and learn what is happening in Denmark on PPIE, and some international articles (n = 6) which had a focus on treatment and care for illnesses identical or similar to what we have experienced as patients and relatives [1–11]. The selected articles mostly described the involvement well and thoroughly. We found that the researchers had done a lot of work and focused on hearing what patients needed. Thus, it was difficult for some of us in the group to critically reflect on the researchers’ work, because we don’t know what challenges they faced or what considerations were behind the choices they made.

We would like to comment on the themes training and recruitment as reported in the articles. Pomey et al. [4] mentioned the importance of the *right* recruitment process and patient match, and Miah et al. [1] described not having minority groups represented in their study as a weakness. We believe it is important that researchers consider carefully how they can find PRs that have true lived experience within the area of research as well as taking care to seek diversity. Many of the groups were homogeneous, and we believe that a group must be diverse to deliver different views. If researchers would look for more diversity, they need to be more flexible in meeting time and place and recruitment processes. We believe it is important to consider whether all PRs need the same training—different people will also have different needs for training.

We also found that the selected articles described a power difference between PRs and researchers; it is important to be aware of the power balance between researchers and PRs. Lindblom et al., described that the PRs felt inferior to the health professionals during the research process [11] and Pomey et al. described how PRs were more comfortable having meetings without the researchers [4]. We consider the number of PRs partaking in a project as crucial in this matter. We think that the fewer PRs involved in a project, the more training and support they will likely need to be able to contribute on equal terms.

Researchers can inadvertently get the reply they are looking for if the PRs are only consulted briefly in one part of the research project. In the 11 articles we read, when researchers and PRs worked in the same group, a reciprocal learning dynamic was described. Therefore, having workshops or meetings with only patients or only researchers, it seems much learning between the two could be lost.

Some of us found it challenging to read the articles, due to reading academic papers in a foreign language and being unfamiliar with the structure of research articles. We had made a support tool for what to look for in the articles, but even then, we needed considerable time as we had to go over the articles several times. It has been interesting to get an insight into how eager and engaged researchers were to involve and collaborate with PRs. We would like future articles to report more on the outcomes of the involvement as experienced by the PRs: what do they gain from the different partnerships?

## Discussion

We searched for papers that reported on researcher and PR partnerships as part of their description and or evaluation of the PPIE process. We found 3667 hits, briefly described the 70 eligible papers for inclusion, and performed a more in-depth analysis of the partnerships using the Involvement Matrix on 40 papers. We see that researchers predominantly took on the role of decision-maker and the PPIE tasks were often described as predetermined by the researchers. As such, researchers defined the PRs’ role in most partnerships. Some of the articles pointed out that this was important to mention at onset and clear role boundaries were perceived as positive by researchers and PRs. The yielded outcomes of PPIE were described as positive, often growing beyond initial expectations; so perhaps the full potential of a partnership is hard to reach when setting clear boundaries from the beginning. The most frequently described enabler was PR training, and both researchers and PRs felt training increased their ability to contribute. Others found offering systematic PR training builds patient capacity for engagement and helped legitimize their role [99]. Green et al. found that when members of the public fulfilled a designated role, they needed training and other support to equip them for this role and fit in a preexisting research structure [100]. Jones and Pietilä (2020) report how this results in PRs aligning themselves with health care professionals and adopting professional language to obtain legitimacy [75]. Our findings suggests, as per existing studies, that often what PRs bring to research projects are *filtered* lived experiences, tailored to meet dominant hermeneutical framework and adjusted to be compatible with existing research structures. Generally, our findings suggest that involved PRs were a homogenous group, and that active steps were taken to make sure that the PRs were *pleasant* collaborators with a constructive attitude who could express themselves clearly and simply. We need to be mindful that if only a selected group of patients are heard sharing *filtered* lived experiences, we risk excluding other perspectives whilst additionally creating a new norm(al) which will alienate other patients from their own (and perhaps different) experience [101], contributing to what Miranda Fricker [102] labels as hermeneutical injustice in PPIE. PPIE has the potential to decrease epistemic injustices in health care by helping mutual understanding between clinicians and patients [103], care should be taken to find breath in the PRs invited and allow them to contribute without having to make attempts to fit the hermeneutical and structural framework of the research world.

Overall, all articles reported positive about their PPIE practices which could indicate a reporting bias as negative experiences may be less pleasant to acknowledge, formulate and publish. Others have published in the past on potential negative consequences of PPIE such as abandonment of research ideas [104], and we believe it is important to continuously have honest conversations about both negative and positive aspects of PPIE. For example, are there cases where PPIE does not lead to changes, and if so, how do we as researchers navigate informing PRs (and funders) about this? Some researchers were concerned for the quality of the research and the opinions of fellow researchers when PRs were to be involved. Others have examples of researchers who omitted that PRs were involved out of fear of having the project rejected [100]. In this review, new knowledge and sharing knowledge were the most frequently reported positive outcomes of PR partnerships, and articles reported that PPIE improved the overall quality. Hence, concerns about experiential knowledge negatively impacting a project seem unwarranted and could benefit a more detailed analysis in the future.

Our analysis showed that when PRs were co-authors, they were more frequently described as partners through most steps of the research cycle and in this way, co-authorship legitimized the PR contribution. There is sparse literature addressing authorship in PPIE research; Richards et al., offered co-authorship as a way of giving credit to someone who made “important intellectual contributions” in a co-production process [105]. Despite of rigorous definitions of co-authorship (i.e. by ICMJE [95], co-authorship attribution is not always in line with these guidelines.. In this article we used PR co-authorship as a variable in our analysis; this provided us with insight into the PPIE practices behind PR-co-authored articles. Our findings showed that PRs earned their authorship by being partners in most parts of the research process.

In a review of reviews, Hoekstra et al. [80] found that partnership descriptions largely depended on research area and country origin of first author. We had similar findings and saw no clear association between PPIE labels or frameworks used and actual partnership practices. This review showed the importance of communication and personal skills, and as per existing literature, these findings suggest that to understand the gains of PPIE activities/practices, we cannot look solely at labels or frameworks; we must also investigate interpersonal relationships and partnership dynamics. As reported, both researchers and PRs can be “strong or difficult personalities” [58]; illustrative of how interpersonal skills are perhaps paramount these elements were only reported as recruitment criteria for PRs and not as a theme for training of researchers. Interpersonal skills of researchers should be an area for future focus.

### Strengths and limitations

This review offers an overview of 70 articles reporting on PPIE activities as well as in depth analysis of a selection of those. The Involvement Matrix may not provide a full representation of partnership roles, as a few articles couldn’t be mapped on the matrix as they lacked thorough descriptions of the PR-involvement throughout all stages. This may have skewed the mapping results, but the mapped papers showed a trend which we feel is representative of our data.

The format of a rapid review is intended to explore the current trends and knowledge on a subject. It is meant to be a quick process (commonly less than 6 months [83]) to assess current knowledge about a policy or practice [82]. Perhaps as reporting on PPIE experiences is a relatively new field, with a lack of agreement on key definitions, this review did not unfold as rapid as intended with much scrutiny needed to extract data from each included article. We tried to alleviate work for future reviews by including a ‘patient partner contributions’ section making their contributions clear. PPIE activities are commonly found to be under-reported and under-reflected [106]. We found especially that research purpose and preparatory stages like origin of research idea and development of protocol were generally under-reported. GRIPP 1 and 2 do not provide a uniform reporting style as anticipated in our inclusion criteria. The IMRAD format for journal publications may limit PPIE reporting [107] and more openness to report personal outcomes for both PRs and researchers could perhaps alleviate this. Until this becomes mainstream, a future focus on actual activities and relations rather than terminology can help shed light on outcomes and impact of PPIE in health research.

## Conclusions

In research partnerships between PRs and researchers, researchers most often have decision-making roles, which gives them control of where, when, and how to involve PRs in their projects. As PR-researcher collaborations seem to evolve during the projects, their full potential may not be reached if fully planned from the start. Co-authorship is can be an acknowledgement and legitimization of PR contributions—and should be used as such, yet, currently happens most often when PRs have had the role of partner in several parts of the research cycle even though they might have made a significant contribution to the research. Across a variety of involvement activities and frameworks, common partnership enablers were found; these include training, interpersonal skills, remuneration, time and trust. Reported PPIE outcomes included: overall improved quality of research and new learning for all parties involved. Care should be taken to include a variety of PRs, and consideration of individual PR needs may create the conditions to invite a more varied group of people into health research.

## Supplementary Information

**Additional file 1.** Consisting of appendix A, B, C, D with extra information on search terms, PPIE activities, inclusion and exclusion criteria, alterations from the protocol and further tables and figures supporting the presentation of our findings.

## Data Availability

The protocol for this study is available at: https://doi.org/10.17605/OSF.IO/QMWVK. The datasets generated and analysed during the current study are included in this published article and its supplementary information files.

## Abbreviations

COPD: Chronic obstructive pulmonary disease
GRIPP2: Guidance for reporting involvement of patients and the public
HTA: Health technology assessment
iKT: Integrated knowledge translation
PAR: Participatory action research
PCORI: Patient-centered outcomes research institute
PPIE: Patient and public involvement and engagement
PRO: Patient reported outcomes
PR: Patient and relative
RCT: Randomized controlled trial

## Declarations

### Author information.

AWK is a nurse and PhD Student at the unit of user perspectives and community-based interventions at the University of Southern Denmark. She currently focuses her work on patient and public involvement in health research.

MJR is an Associate Professor in Diabetes Care, Patient and Public Involvement and Technology at Steno Diabetes Center Odense, Odense University Hospital and University of Southern Denmark. She is interested in diabetes care, clinical research and user involvement, and she is acknowledged within the scientific tradition of Participatory design in Health Science.

MK is an Associate Professor in Pediatric Rehabilitation at the University Medical Center Utrecht and De Hoogstraat Rehabilitation in the Netherlands. Her research interests include family-centered services, and the role of the family in the development of children with disabilities. Involving patients and families in all stages and aspects of research is one of the key principles in her research.

AKS is a patient partner in AWK’s PhD project. She is also a patient and relative representative at Odense University Hospital and a patient guest lecturer at University of Southern Denmark.

KB is a patient partner in AWK’s PhD project and serves as a patient representative in several regional councils of Southern Denmark and at Odense University Hospital.

KEB is a patient partner in AWK’s PhD project and serves as a patient representative in several councils at Odense University Hospital.

TA is a partner in AWK’s PhD project and a patient and relative representative in various employment committees at Odense University Hospital. He also occasionally teaches new staff about the experiences of being a relative.

MLK is a PhD student at University of Southern Denmark. She has a basic education as a trained nurse and a master’s degree in health sciences. Her research interest includes existential and spiritual care, missed nursing care and user involvement in healthcare research.

AJ is a Professor in Patient and Public Participation at Utrecht University Medical Center, Utrecht University (Netherlands), and University of Southern Denmark. She is a health service researcher with a passion and longstanding commitment to issues of democratisation, participation and service user involvement in health research and health education. She holds honorary positions with Exeter University Medical School (UK) and Odense University Hospital (Denmark).
