# Supplementary material for: Roles, outcomes, and enablers within research partnerships: A rapid review of the literature on patient and public involvement and engagement in health research
Source: Res Involv Engagem. 2023 Jun 15;9:43. doi: 10.1186/s40900-023-00448-z (PMC10268359; doi:10.1186/s40900-023-00448-z)
Supplement: Supplementary file 1 — Additional file 1. Consisting of appendix A, B, C, D with extra information on search terms, PPIE activities, inclusion and exclusion criteria, alterations from the protocol and further tables and figures supporting the presentation of our findings. [file 40900_2023_448_MOESM1_ESM.docx]

**Additional file 1**

**Appendix A:**

***Table S1: SPIDER tool of search terms and blocks*** (Inspired by Cooke et al. (2012)

| SPIDER TOOL | Search terms |
| --- | --- |
| Sample | Patient*, relative*, public, user, researcher*, consumer*, caregiver*, stakeholder. |
| Phenomenon of Interest | Participat*, involv*, engag*, collaboration, coproduction, co-production, cocreation, co-creation, codesign, patient reported outcomes, inclusion, citizen science. |
| Design | Design, Interview, focus group, case stud*, observ*, ethno*, narrative*, evaluation. |
| Evaluation | Experience*, value creation, value, partner*, role*, knowledge creation, knowledge sharing, legitimacy, relation*. |
| Research Type | Health research, health services research |

***Figure S1: Medline search string***

| 1 | Patient participation/ |
| --- | --- |
| 2 | Consumer participation/ |
| 3 | (patient* or relative*or public or user or researcher*or consumer*or caregiver*or stakeholder) ti.ab.kf. |
| 4 | 1 or 2 or 3 |
| 5 | (participat*or involv*or engag*or collaboration or coproduction or co-production or cocreation or co-creation or codesign or patient reported outcomes or inclusion or citizen science) ti.ab.kf. |
| 6 | (design or Interview or focus group or case stud* or observ* or ethno*or narrative*or evaluation) ti.ab.kf. |
| 7 | (experience* or value creation or value or partner* or role* or knowledge creation or knowledge sharing or legitimacy or relation*) ti.ab.kf. |
| 8 | Health services research/ |
| 9 | Health research.mp |
| 10 | 8 or 9 |
| 11 | 4 and 5 and 6 and 7 and 10 |
| 12 | Limit 11 to yr=”2012-Current” |

**Appendix B**

**GRIPP2 Short form**

| Section and topic | Item | Reported on page No |
| --- | --- | --- |
| 1: Aim | To allow both experiential and scientific knowledge to inform the study.  The topic is PPIE and it is a democratic right for patients and relatives to be part of improving our knowledge on the topic. Authors (AWK, MJR, AJ are affiliated with Odens University Hospitalm where a new strategy of involving patients and relatives in research has been put in place. Therefore it is paramount to include them in building our knowledge base on patient and public involvement. | 3 |
| 2: Methods | We have used the Involvement Matrix as a guide for our involvement activities: develop research focus (AKS), analysis strategy (AKS), data extraction and analysis (AKS, KEB, KB, TA), reflection and discussions of findings (AKS, KEB, KB, TA). This was mostly done via group meetings and one on one meetings between AWK and PRs with the option of email and phone conversations which resulted in them continuously being part of the entire process. They have written a section of the paper ‘Patient partners’ observations’. We did not use an already developed “training pack”, but together decided on an iterative approach offering support where and when the individual needed it. At the time of conducting the analysis the group was familiar with each other and reciprocal trust in voicing needs and concerns had been established. | 4  16 |
| 3: study results | The study has had focus on both patients and researcher roles, which was suggested by the PRs. Therefore, the outcomes of this study were changed according to PR input. The discussions the PRs and researchers have had formed the paper to include a wider understanding and richer analysis of both the patients and the researcher perspective.  It was hard work for the PRs to read scientific articles and to take a critical stance towards the included papers. This meant that some activities were not done as a group but rather on a one on one basis. | 16 |
| 4: Discussion and conclusions | The discussions are more detailed with several viewpoints discussed. PRs influenced the whole study as they were part of developing all aspects of it.  During discussions it became clear, that their observations deserved a dedicated section to make it clear what PRs see as important in the articles. | 17  16 |
| 5: Reflections/  critical perspective | The research team and PRs had the initial same ideas for this study. The PRs developed the focus to include both patients and researchers - something which has improved the study tremendously. PRs also were part of developing the analytic matrix which made the analysis more focused on conditions for PRs when involved.  If we were to do the study again, we would have spent extra care to explain how and support the PRs in reading the scientific articles. Some found it very difficult, and we could have alleviated that with more support. This would have required additional finances and resources for example by using a professional translations services rather than getting by with Google translate. The fact that the first author and the PR group had established a relation at the start of the project helped in understanding where or how the individual needed support. However, researchers must take extra care continuously to “check” whether additional needs for support appear when PRs are charged with tasks to perform at home between meetings. | Protocol  4 and GRIPP2 |

**Patient Partner Panel and PhD-student role in this rapid review via the Involvement Matrix.**

| Stage | | Listener  (is given information) | Co-thinker  (is asked to give opinion) | Advisor  (gives (un)solicited advice) | Partner  (works as an equal partner) | Decision-maker  (takes initiatives and/or decisions) | |  |
| --- | --- | --- | --- | --- | --- | --- | --- | --- |
| Preparation | **Research question** |  |  |  | **X X** |  |  |  |
|  | **Protocol** |  |  | **X** |  | **X** |  |  |
|  | **Study design** |  |  |  | **XX** | **X** |  |  |
| EXECUTION | **Data collection** |  | **X** |  |  | **X** |  |  |
|  | **Data analysis** |  |  |  | **XX** |  |  |  |
| IMPLEMENTation | **Write-up** |  |  |  | **XX** |  |  |  |
|  | **Dissemi-nation** |  |  |  | **(XX)** |  |  |  |
|  | ***PR role in BLUE, researcher role in Dark blue***  **(In brackets: Not commenced yet)** | | | | | | | |

**Appendix C – Inclusion and exclusion criteria as depicted in the protocol for this review**

**(**Karlsson A, Kragh-Sørensen A, Rothmann M, Ketelaar M, Janssens A. Partnerships within coproduced research - a rapid review protocol. 2021. Published on OSF: DOI:10.17605/OSF.IO/QMWVK).

Study selection

|  | Include | Exclude |
| --- | --- | --- |
| Study design | Qualitative Interviews/ questionnaires/ observations, discourse analysis, narrative, grounded theory, thematic analysis.  Ethnographic studies exploring PR-researcher interaction/relationships.  Qualitative and Quantitative studies involving PPI as part of the research.  Case studies | Historic or theoretical focus on coproduction.  Articles focusing on mere classification of involvement.  Qualitative or Quantitative studies not reflection on PPI involvement in their study.  Reviews of coproduction literature.  Protocols  Studies focusing on involvement in treatment and care. |
| Population | Patients/ relatives /researchers | Trial subjects |
| Setting | Health research | Social services, development work, climate research. |
| Intervention | Studies reporting on evaluation of coproduced research / the involvement process in research. | Studies focusing on patient involvement in the clinical /practical field – e.g., shared decision making. |
| Outcome | Evaluation of the coproduction process e.g., in a GRIPP 1 (1) or GRIPP 2 (2) form. |  |
| Publication Status | English-language  Peer-reviewed journal or reports  Publication year 2012 onwards |  |
|  | **Limit** | **Impact** |
| Steps removed from Systematic Review process | Limit number of searched databases to 4. Exclude grey literature search.  Limit bias analysis  Exclude quality assessment  Create search strings with high specificity.  Limit Publication years to 2012 onwards when the first PI guidelines on reporting were published (GRIPP 1) | This will likely limit the number of reports and non-scholary articles. This may result in fewer results for screening, and this is part of the rapid screening strategy.  Unless there are strong indications of certain biases, they are unlikely to affect the results of this review but will of course have an impact on the quality of the individual studies included.  For the scope of this study, a quality assessment of the study intervention will not add value to the results of this review and will therefore be omitted.  To retrieve a manageable screening list as well as suiting the rapid character of this review, a high specificity has been attempted by including 5 search blocks as recommended by the spider tool for qualitative searches. It is likely that some relevant articles may be missed due to this, but in the trial searches a choice of manageable screening numbers had to be made. To compensate slightly, the keywords and term search will be attempted to be near exhaustive.  According to a recent bibliometric review, the number of articles reporting on PPI doubled between 2014-2015 and the main body of literature (80% of the retrieved documents) on coproduction is written after 2018 (Fusco et al 2020). A good example of this is how Price et al (2018) found that in 2013-14, before the BMJ introduced their PPI reporting policy, 1 out of 189 published papers reported PPI activity (0.5%). After the policy was introduced in 2015 16 out of 152 (11%) published research papers reported PPI activities (3). The choice of excluding literature from before 2012 has therefore been made as it seems the main body of literature is to be found after and will perhaps be more comparable as the development of guidelines in the form of GRIPP 1 (and later 2) came in 2011 (1). |

*Abbreviations: PR = patient and relative, PPI = patient and public involvement, GRIPP = Guidance for Reporting Involvement of Patients and the Public.*

**Alterations to the protocol**

Data extraction: In the protocol we planned that two researchers screened 50% of the of search results independently. At midway comparison for disagreement, disagreement was at 15% which was well below the expected 30%. After discussion with referee Jacob Dennis Larsen, we decided to allow further screening to be done by one researcher as this would save considerable time and resources.

Synthesis: As described in the main article, a much larger number of articles than anticipated was found. Therefore, it was decided to select articles for detailed analysis, but to keep inclusion and exclusion criteria as stated in the protocol. The resulting list of 70 articles can serve as a resource for others in need of PPIE evaluations in specific research fields.

## References

1. Staniszewska S, Brett J, Mockford C, Barber R. The GRIPP checklist: Strengthening the quality of patient and public involvement reporting in research. International Journal of Technology Assessment in Health Care. 2011;27(4):391-9.

2. Staniszewska S, Brett J, Simera I, Seers K, Mockford C, Goodlad S, et al. GRIPP2 reporting checklists: tools to improve reporting of patient and public involvement in research. BMJ. 2017:j3453.

3. Price A, Schroter S, Snow R, Hicks M, Harmston R, Staniszewska S, et al. Frequency of reporting on patient and public involvement (PPI) in research studies published in a general medical journal: a descriptive study. BMJ Open. 2018;8(3):e020452.

**Appendix D:**

**Table S2: visualization of results**

**Articles grouped by research design and research area**

| 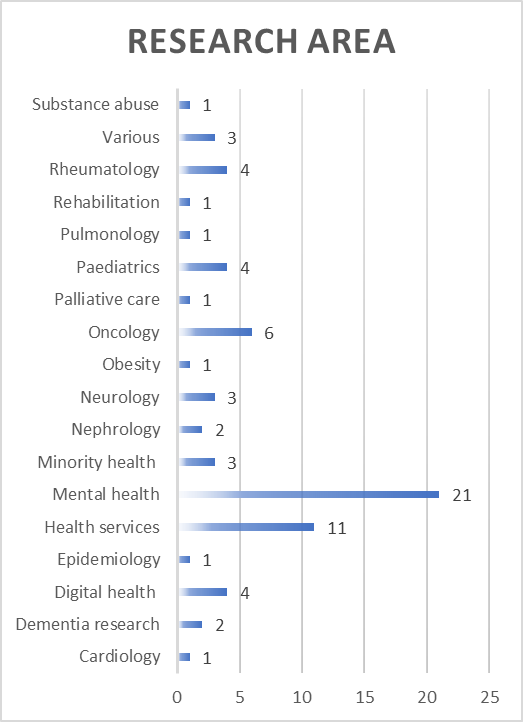 | 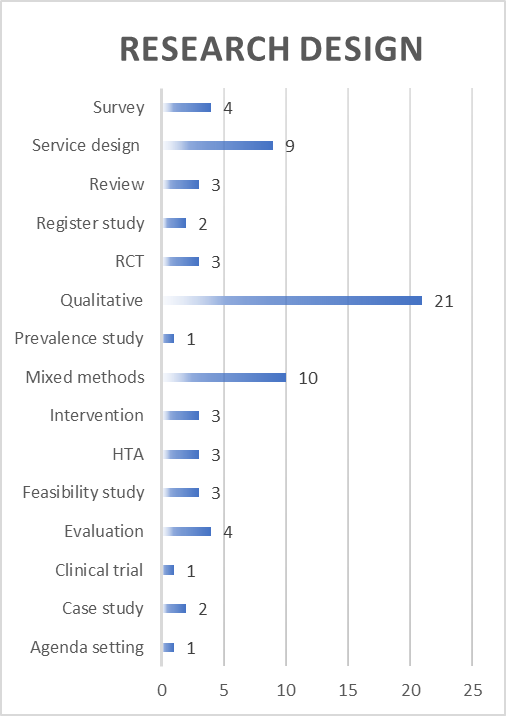 |
| --- | --- |

*Note: Health services research defined here as:” Inquiry to produce knowledge about the structure, processes, or effects of personal health services” (National Research Council. Health Services Research: Report of a Study. Washington, DC: The National Academies Press; 1979).*

***Table S3: PR and Researcher roles analysed with Smits et al.’s Involvement Matrix – data details***

| Stage | | Listener  (is given information) | Co-thinker  (is asked to give opinion) | Advisor  (gives (un)solicited advice) | Partner  (works as an equal partner) | | Decision-maker  (takes initiatives and/or decisions) | |
| --- | --- | --- | --- | --- | --- | --- | --- | --- |
| Preparation | **Research question** | **Alexander, 2021**  **Beighton, 2019**  **Burrows, 2019**  **Jørgensen, 2018**  **Lindblom, 2021**  **Nissen, 2018**  **Nöstlinger, 2016**  **Marks, 2018**  **Nierse, 2012** | **Jewell, 2019** | **Chiu, 2013**  **Bourque, 2020 Dawson, 2020**  **Gammon, 2014**  **Hoekstra 2021,**  **Worsley, 2021** | **Chiu, 2013**  **Hutchinson, 2013**  **Simpson, 2018**  **Carr, 2019** | | **Alexander, 2021**  **Beighton, 2019**  **Burrows, 2019**  **Chiu, 2013**  **Cook, 2019**  **Devonport, 2018**  **DeWit, 2015**  **Frankena, 2019**  **Froggatt, 2016**  **Hitchen, 2014**  **Jewell, 2019**  **Jørgensen, 2018**  **Kearns, 2020**  **Melchior, 2021**  **Lindblom, 2021** | **Miah, 2020**  **Nissen, 2018**  **Nöstlinger, 2016**  **Ostrach, 2021**  **Rayment, 2017**  **Simpson, 2018**  **Bourque, 2020**  **Carr, 2019**  **Dawson, 2020**  **Gammon, 2014**  **Hoekstra, 2021**  **Marks, 2018**  **Nierse, 2012**  **Sharmil, 2021** |
|  | **Protocol** | **Burrows, 2019**  **Lindblom, 2021**  **Nissen, 2018**  **Nöstlinger, 2016** | **Chiu, 2013**  **Jewell, 2019**  **Marks, 2018** | **Alexander, 2021, Chiu, 2013**  **Jørgensen, 2018**  **Miah, 2020**  **Rayment, 2017**  **Vogsen, 2020** | **Chiu, 2013**  **Hutchinson, 2013**  **Simpson, 2018**  **Gammon, 2014** | | **Alexander, 2021**  **Devonport, 2018**  **Hitchen, 2014**  **Jewell, 2019**  **Jørgensen, 2018**  **Kearns, 2020**  **Lindblom, 2021**  **Miah, 2020**  **Nissen, 2018 Nöstlinger, 2016** | **Rayment, 2017**  **Seeralan, 2020**  **Simpson, 2018**  **Gammon, 2014**  **Marks, 2018,**  **Vogsen, 2020**  **Worsley, 2021**  **Sharmil, 2021** |
|  | **Study design** | **Alexander, 2021**  **Beighton, 2019**  **Burrows, 2019**  **Sharmil, 2021** | **Cook, 2019**  **Nöstlinger, 2016**  **Marks, 2018** | **Jewell, 2019**  **Jørgensen, 2018**  **Lindblom, 2021**  **Nissen, 2018**  **Nissen, 2018**  **Simpson, 2018**  **Hoekstra, 2021**  **Hoekstra, 2021**  **Skovlund, 2020**  **Vogsen, 2020**  **Worsley, 2021** | **Chiu, 2013**  **Hitchen, 2014**  **Hutchinson, 2013**  **Kearns, 2020**  **Kearns, 2020**  **Ostrach, 2021**  **Ostrach, 2021**  **Seeralan, 2020**  **Seeralan, 2020** | **Bourque, 2020**  **Bourque, 2020**  **Carr, 2019**  **Carr, 2019**  **Gammon, 2014 Gammon, 2014 Hoekstra, 2021 Hoekstra, 2021**  **Nierse, 2012**  **Nierse, 2012**  **Vat, 2020**  **Vat, 2020** | **Alexander, 2021**  **Beighton, 2019**  **Burrows, 2019**  **Chiu, 2013**  **Cook, 2019**  **Devonport, 2018**  **DeWit, 2015**  **Frankena, 2019**  **Froggatt, 2016**  **Hitchen, 2014**  **Jewell, 2019**  **Jørgensen, 2018** | **Lindblom, 2021**  **Miah, 2020**  **Nissen, 2018**  **Nöstlinger, 2016**  **Rayment, 2017**  **Simpson, 2018 Hoekstra, 2021**  **Honey, 2019**  **Marks, 2018**  **Sharmil, 2021**  **Skovlund, 2020**  **Vogsen, 2020**  **Worsley, 2021** |
| EXECUTION | **Data collection** | **Beighton, 2019**  **Burrows, 2019**  **Hoekstra, 2021**  **Marks, 2018**  **Pomey, 2020**  **Vogsen, 2020** | **Jewell, 2019**  **Nissen, 2018** | **Alexander, 2021**  **Chiu, 2013**  **Lindblom, 2021**  **Rayment, 2017**  **Honey, 2019** | **Chiu, 2013**  **Froggatt, 2016**  **Froggatt, 2016**  **Jørgensen, 2018**  **Nöstlinger, 2016**  **Ostrach, 2021**  **Ostrach, 2021**  **Seeralan, 2020**  **Seeralan, 2020** | **Bourque, 2020**  **Bourque, 2020**  **Gammon, 2014**  **Gammon, 2014, Hoekstra, 2021**  **Nierse, 2012**  **Nierse, 2012**  **Sharmil, 2021**  **Sharmil, 2021** | **Alexander, 2021**  **Beighton, 2019**  **Burrows, 2019**  **Chiu, 2013**  **Cook, 2019**  **Devonport, 2018**  **DeWit, 2015**  **Frankena, 2019**  **Hutchinson, 2013**  **Jewell, 2019** | **Jørgensen, 2018**  **Kearns, 2020**  **Miah, 2020**  **Nissen, 2018**  **Rayment, 2017**  **Simpson, 2018**  **Hoekstra, 2014**  **Honey, 2019 Pomey, 2020**  **Vogsen, 2020**  **Worsley, 2021** |
|  | **Data analysis** | **Beighton, 2019**  **Burrows, 2019**  **Nissen, 2018** | **Chiu, 2013** | **Jewell, 2019**  **Hoekstra, 2021**  **Honey, 2019**  **Marks, 2018**  **Vogsen, 2020** | **Froggatt, 2016**  **Froggatt, 2016**  **Hitchen, 2014**  **Hitchen, 2014**  **Jørgensen, 2018**  **Melcior, 2021**  **Melcior, 2021**  **Nöstlinger, 2016**  **Nöstlinger, 2016**  **Ostrach, 2021**  **Ostrach, 2021**  **Seeralan, 2020**  **Seeralan, 2020**  **Bourque, 2020**  **Bourque, 2020**  **Carr, 2019** | **Carr, 2019**  **Gammon, 2014**  **Gammon, 2014**  **Hoekstra, 2021**  **Hoekstra, 2021**  **Nierse, 2012**  **Nierse, 2012**  **Vat, 2020**  **Vat, 2020**  **Sharmil, 2021**  **Sharmil, 2021**  **Skovlund, 2020,**  **Skovlund, 2020**  **Vogsen, 2020**  **Vogsen, 2020** | **Alexander, 2021**  **Beighton, 2019**  **Burrows, 2019**  **Chiu, 2013**  **Cook, 2019**  **Devonport, 2018**  **DeWit, 2015**  **Frankena, 2019**  **Froggatt, 2016**  **Jewell, 2019** | **Jørgensen, 2018**  **Kearns, 2020**  **Lindblom, 2021**  **Miah, 2020**  **Nissen, 2018**  **Rayment, 2017**  **Simpson, 2018 Hoekstra, 2021 Honey, 2019**  **Marks, 2018**  **Vat, 2020**  **Worsley, 2021** |
| Implementation | **Write-up** | **Beighton, 2019**  **Burrows, 2019** | **Alexander, 2021**  **Miah, 2020** | **Hoekstra, 2021**  **Marks, 2018**  **Vogsen, 2020**  **Worsley, 2021** | **Jørgensen, 2018 Bourque, 2020**  **Bourque, 2020**  **Carr, 2019**  **Carr, 2019**  **Dawson, 2020**  **Dawson, 2020**  **Faulkner, 2021**  **Gammon, 2014** | **Gammon, 2014**  **Nierse, 2012**  **Nierse, 2012**  **Sharmil 2021**  **Sharmil, 2021**  **Skovlund, 2020**  **Skovlund, 2020** | **Alexander, 2021**  **Beighton, 2019**  **Burrows, 2019**  **Chiu, 2013**  **Cook, 2019**  **Devonport, 2018**  **DeWit, 2015**  **Frankena, 2019**  **Froggatt, 2016**  **Hitchen, 2014**  **Jewell, 2019**  **Jørgensen, 2018** | **Kearns, 2020**  **Lindblom, 2021**  **Miah, 2020**  **Nissen, 2018**  **Nöstlinger, 2016**  **Rayment, 2017**  **Simpson, 2018**  **Faulkner, 2021**  **Hoekstra, 2021**  **Marks, 2018**  **Vogsen, 2020**  **Worsley, 2021** |
|  | **Dissemina-tion** | **Beighton, 2019**  **Burrows, 2019** | **Alexander, 2021** | **Chiu, 2013** | **Chiu, 2013**  **Hutchinson, 2013**  **Jørgensen, 2018**  **Bourque, 2020**  **Bourque, 2020**  **Carr, 2019**  **Carr, 2019**  **Dawson, 2020**  **Dawson, 2020**  **Faulkner, 2021** | **Gammon, 2014**  **Gammon, 2014**  **Marks, 2018**  **Marks, 2018**  **Skovlund, 2020**  **Skovlund, 2020**  **Sharmil, 2021**  **Sharmil, 2021**  **Vogsen, 2020**  **Vogsen, 2020** | **Alexander, 2021**  **Beighton, 2019**  **Burrows, 2019**  **Chiu, 2013**  **Cook, 2019**  **Devonport, 2018**  **DeWit, 2015**  **Frankena, 2019**  **Froggatt, 2016**  **Hitchen, 2014**  **Jewell, 2019** | **Jørgensen, 2018**  **Kearns, 2020**  **Lindblom, 2021**  **Miah, 2020**  **Nissen, 2018**  **Nöstlinger, 2016**  **Rayment, 2017**  **Simpson, 2018**  **Faulkner, 2021 Hoekstra, 2021 Sharmil, 2021**  **Worsley, 2021** |
|  | ***Non-co-author Articles: PR role in BLUE, researcher role in DARK BLUE***  ***Co-author Articles: PR role in RED, Researcher role in DARK RED*** | | | | | | | |
